# Diffusion and Controlled Release in Physically Crosslinked Poly (Vinyl Alcohol)/Iota-Carrageenan Hydrogel Blends

**DOI:** 10.3390/polym12071544

**Published:** 2020-07-13

**Authors:** Catalin Croitoru, Ionut Claudiu Roata, Alexandru Pascu, Elena Manuela Stanciu

**Affiliations:** Materials Engineering and Welding Department, Transilvania University of Brasov, Eroilor 29 Str, 500036 Brasov, Romania; alexandru.pascu@unitbv.ro (A.P.); elena-manuela.stanciu@unitbv.ro (E.M.S.)

**Keywords:** poly (vinyl alcohol), iota-carrageenan, polymer blends, polysaccharide, hydrogels, swelling, controlled release

## Abstract

This paper reports the obtaining of poly (vinyl alcohol) and ι-carrageenan blend hydrogels by physical crosslinking (consecutive freeze–thaw cycles). The two polymers were completely miscible in the weight ratio interval used in this study, as determined by solution viscometry data. Strong interactions through hydrogen bonding and forming of mixed interpolymer crystalline domains were observed, which are responsible for the formation of stable drug release-tunable matrices. The release profiles of three model antibiotic drugs (amoxicillin, tetracycline hydrochloride, and gentamicin sulfate) were assessed in a pH interval between 3 and 7.3. They were found to be strongly dependent on the drug chemistry, mesh size of the hydrogels, swelling mechanism, and pH of the release medium. A decrease of up to 40% in the release rates and up to 10% in the diffusion coefficients of the model drugs was registered with the increase in ι-carrageenan content.

## 1. Introduction

Poly (vinyl alcohol) (PVA) has been widely in used in the formulation of hydrogel matrices for controlled-release or environmental remediation applications since the early 1960s, due to its nontoxicity, biocompatibility, and to the availability of an extensive palette of chemical modification (functionalization) and crosslinking reactions [1,2]. PVA chemical modification and crosslinking represent one of the two primary tools through which the solubility, porosity, diffusion, swelling, sorption yield, and hydrophilicity of PVA hydrogels could be tuned to match various applicative demands from both research and industry [3,4]. The second method to tune the properties of PVA hydrogels (applied either standalone or in combination with the first) is represented by PVA compounding with various inorganic or organic compounds [5,6], respectively, blending with synthetic and/or natural polymers [7,8].

Blending PVA with biopolymers (especially polysaccharides) increases the biocompatibility, hydrophilicity, and swelling of hydrogels, through the increase in the density of the hydrophilic groups [9,10]. It also improves the flexibility, hardness, and compression resistance of the PVA-blend hydrogels through the creation of extended crosslinking points in the polymer matrix [11]. Moreover, the addition of polysaccharides with ionized or ionizable groups to PVA, for example chitosan, alginates, carrageenan, pectin, pectinates, or modified cellulose, leads to the formation of hydrogels with new functional properties (antimicrobial materials [12], sensors [13]). Supplementary, this addition modulates the release of ionic or polar active principles (antibiotics, nutraceuticals, and other drugs) in controlled-release applications, while also extending or improving the performance of the PVA hydrogel matrix in conjunction with the sorption of potentially harmful species (e.g., dyes [14], heavy metal cations [15], anions [16], and pesticides [17]) for environmental remediation applications.

Carrageenans represent a class of linear water-soluble sulfated galactan polysaccharides that are isolated mainly from marine red algae. This class of biopolymers possesses a high tendency to form thermoreversible gels through extended hydrogen bonding between molecules adopting a single-helix conformation (λ) or between double-helix molecular associations (κ and ι carrageenans) [18,19]. Carrageenans form strong interpolymer complexes through hydrogen bonding with poly (vinyl alcohol), these complexes imparting higher tensile strength and improved water barrier properties to PVA films in comparison with other biopolymers such as Na-alginate, gelatin, chitosan or carboxymethylcellulose [20]. Carrageenan (CAR) is entirely miscible with poly (vinyl alcohol) in the amorphous region, while in the crystalline domain miscibility is only partial, due to differences in the conformation and molecular weight between the two polymers [21,22]. PVA/CAR hydrogels crosslinked through the application of β and γ radiation or alternative freeze–thaw cycles (physical crosslinking) have been found useful for wound dressing [11,23] or cultivation of microalgae [24], and in the lyophilized (freeze-dried) state, also suitable for tissue engineering applications [25]. Ionic-crosslinked and chemically crosslinked PVA/κ-carrageenan hydrogels and films have been found to possess excellent in vivo biocompatibility and functionality as drug release vehicles [26,27]. Composite PVA/κ-carrageenan hydrogel matrices obtained through physical crosslinking have also been applied as sorbents for cationic dyes [28] or as support for photocatalytic oxides [29] and bacteria [30] in environmental remediation applications. 

In this paper, poly (vinyl alcohol) has been blended with ι-carrageenan in different weight ratios to obtain hydrogels through physical crosslinking (alternative freeze–thaw cycles). The hydrogels were used as drug release vehicles for three types of antibiotics: tetracycline, amoxicillin and gentamicin sulfate. To the best of our knowledge, this would be the first report of using ι-carrageenan as a coblending component to obtain hydrogels through physical crosslinking. The benefit of using this carrageenan stems from the presence of two sulfate groups per galactan structural unit, which allows for increased interaction with PVA through hydrogen bonding, and more active binding/release tuning sites in the hydrogel matrix, compared to κ-carrageenan which presents one sulfate group per the same structural unit. Additionally, only a few studies have used ι-carrageenan-containing materials as drug release matrices. This study could serve to enlarge the dynamically expanding database of biopolymer-synthetic polymers systems description, as well as of hydrogel materials application possibilities.

## 2. Experimental

### 2.1. Materials

Poly (vinyl alcohol) (average M_w_ of 130,000; 99.4 % hydrolysis degree), ι-carrageenan (dynamic viscosity of 55 mPa⋅s at 75 °C in 1.5 wt % aqueous solution), κ-carrageenan (dynamic viscosity of 15 mPa⋅s, 0.3% in H_2_O at 25 °C), tetracycline hydrochloride (C_22_H_25_ClN_2_O_8_, ≥95%, coded with T) amoxicillin (C_16_H_19_N_3_O_5_S, potency ≥ 900 μg per mg, coded A), gentamicin sulfate (C_19_H_40_N_4_O_10_S, potency: ~600 μg per mg, coded G), and cyclohexane (anhydrous, 99.5%) were purchased from Sigma-Aldrich (Darmstadt, Germany) and used without further purification.

The reagents used for spectrophotometric determination of the antibiotics (ascorbic acid and ninhydrin) were purchased from the same company.

### 2.2. Hydrogels Obtaining

Four types of hydrogels were prepared in this study, corresponding to poly (vinyl alcohol), ι-carrageenan, respectively, to two PVA and CAR blends, with their composition and sample coding reflected in Table 1. These ratios were chosen considering our hydrogel recipe optimization trials and our previous study on PVA/κ-carrageenan hydrogels [28], which were more dimensionally and compositionally stable up to a 0.140 κ-carrageenan weight fraction.

The starting 10 wt/vol % poly (vinyl alcohol), respectively, 2 wt/vol % ι-carrageenan aqueous solutions were prepared through dispersing the required amount of polymer in distilled water, followed by magnetic stirring of the resultant disperse system at 90 °C for 5 h until homogenization, followed by filtering through a 1 μm stainless steel wire mesh and cooling to room temperature (21 ± 1 °C). The PVA and CAR mixtures were prepared by magnetically stirring the required volume of the two polymer solutions at 90 °C for 30 min. For hydrogels obtaining, 10 mL of PVA, CAR, and PVA:CAR solution mixtures were cast into borosilicate Petri dishes (60 mm diameter) and submitted to five consecutive cycles of freezing and thawing. The freezing temperature was –25 °C, the freezing duration 12 h, while the thawing temperature was 22 °C, with a corresponding thawing duration of 12 h.

**Figure 1:**
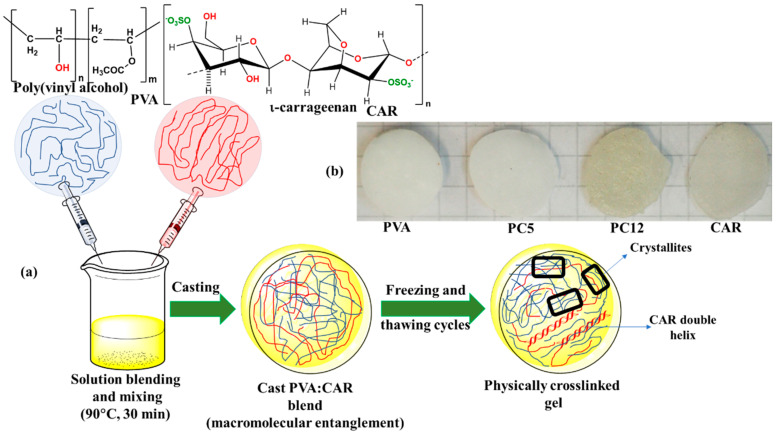
(**a**) Preparation steps for the physical-crosslinked hydrogels; (**b**) photographic image of the prepared hydrogels.

The molecular structure of poly (vinyl alcohol), ι-carrageenan, the work steps involved in the preparation of the gels, and in the physical crosslinking mechanism is illustrated in Figure 1.

The antibiotic-loaded hydrogels were prepared through applying the same freeze–thaw procedure as for the neat hydrogels, adding a specified amount of drug (*m_drug_*) per 10 mL of PVA and respectively, PVA:CAR solution mixtures, followed by stirring at 35 °C to avoid thermal degradation, until complete dissolution of the antibiotics. The amount of drug added in each of the formulations corresponding to Table 1 was kept at 250 mg/g of dry polymer(s). Since the carrageenan gel alone did not show satisfactory stability to water action, an antibiotic-loaded gel was not prepared for this type of material. The antibiotic-loaded hydrogels coding is preserved from the neat samples, with adding “-T”, “-A”, and “-G” as a suffix, for tetracycline hydrochloride, amoxicillin, respectively, gentamicin sulfate.

The obtained neat hydrogels were stored in distilled water (in swollen equilibrium state) before preparing the samples for the structural and morphological analyses detailed in the following subsections.

For the sake of comparison, κ-carrageenan containing hydrogels were prepared with the corresponding composition of PC12 (6 mL of 10 wt % PVA solution and 4 mL of 2 wt % κ-carrageenan solution, *w_CAR_* = 0.117), following an identical protocol. These hydrogels were coded with PC12K. The same blend composition, prepared with κ-carrageenan, was loaded with tetracycline hydrochloride, to assess the differences between the delivery profiles of the two carrageenans (gel coded with PC12K-T).

### 2.3. Hydrogels Characterization

#### 2.3.1. Solids Content and Gel Content Determination

The solids content, i.e., the percentual dry weight (*SC*, %) of the hydrogels was determined as the percentage ratio between the mass of the oven-dried gels (105 °C, 5 h) and the mass of the as-obtained gels before drying (Table 1). The worksteps for determining the gel content (GC) are described in detail in our previous research [28]. Three determinations were employed for both the solids content, respectively. The gel content for each type of material, and the average values were presented in Table 1.

#### 2.3.2. Swelling Behavior

The swelling behavior was studied by immersing preweighted (initial mass *m*_0_) circular disks (~5 mm diameter) of the PVA, PC5, PC12, and CAR hydrogels in 100 mL of distilled water, respectively, and 100 mL of aqueous media with different pH values (between 3 and 7.3) at room temperature. The values of pH were adjusted by the addition of HCl, respectively, NaOH of different concentrations to maintain a constant ionic strength of the swelling media. The values of pH for assessing the hydrogels swelling were chosen to be representative for the control release experiments. The cryogel disks were taken out of the swelling media at determined time intervals, dried at the surface with filter paper, and weighed (*m*_t_), after which they were reimmersed in the same media. These steps were repeated until the gels reached the swelling equilibrium. The swelling degree (*SD*, %) for any given swelling period *t* until equilibrium reaching was calculated with Equation (1)

(1) $$SD\;=\frac{m_{t}-m_{0}}{m_{0}}\cdot 100\;\left(\%\right)$$

The diameter and thickness of the gel samples in the initial state, before swelling (*d*_0_, respectively, δ), as well as the diameter and thickness after 15 min of cumulative immersion in the swelling fluids (*d_sw_*, respectively, *δ_sw_*) were measured with a micrometer (Insize, Zamudio, Spain). The modification in volume after 15 min of swelling *ΔV*_15_ was calculated taking into account the cylindrical form of the samples as $\Delta V_{15}=\pi \cdot {\left(d_{sw}-d_{0}\right)}^{2}\cdot \left(\delta_{sw}-\delta \right)/4$. The swelling experiments and the measurement of thickness and diameter were performed in triplicate for each cryogel, and the average values were presented and discussed in the paper.

Based on the swelling data in distilled water, the amount of crosslinking in the neat and drug-loaded cryogels (the latter corresponding to the PC12 formulation) was determined by calculating the crosslinking density (ν) (Equation (2)), and the number-average molecular weight between crosslinking points (*M_c_*) (Equation (3)), according to the Flory–Rehner theory [31].

(2) $$\nu \;=-\frac{\ln\left(1-\Phi_{s}\right)+\Phi_{s}+\chi \cdot \Phi_{s}^{2}}{\Phi_{s}^{1/3}\cdot V_{0}}$$

(3) $$M_{c}=\frac{d_{h}}{\nu }$$

where ϕ_s_ represents the volume fraction corresponding to the polymer in the swollen hydrogel in the equilibrium state, calculated with Equation (4), *d*_h_ is the density of the hydrogel in the swollen equilibrium state (determined with a solids pycnometer, with cyclohexane as pycnometric fluid; 21 °C), V_0_ is the molar volume of water (18.0360 cm^3^mol^−1^ at 21 °C), and χ is the Flory–Huggins polymer–solvent interaction parameter, calculated according to Equation (5) [31].

(4) $$\Phi_{s}={\left[1+\frac{d_{h}}{d_{H_{2}O}}\cdot \left(\frac{m_{\mathrm{swollen}}}{m_{\mathrm{dry}}}-1\right)\right]}^{-1}$$

In Equation (4), *d*_H2O_ is the density of water (*d*_H2O_ = 0.9893 g/cm^3^ at 21 °C, determined with the pycnometric method), *m*_swollen_ is the mass of the hydrogel in equilibrium swollen state and *m*_dry_ is the mass of dry gel (xerogel), calculated with the value of the solids content (Table 1, *m*_dry_ = *m*_gel_⋅SC/100, where *m*_gel_ is the mass of the as-obtained hydrogel). 

For each type of hydrogel, five different measurements for *m*_swollen_, *m*_dry_ and *d*_h_ have been performed, and the average values were used for calculating ϕ_s_ and subsequently *M*_c_.

The Flory–Huggins polymer–solvent interaction parameter (χ) was calculated with Equation (5) [32]:

(5) $$\chi \;=-\frac{\ln\left(1-\Phi_{s}\right)+\Phi_{s}}{\Phi_{s}^{2}}$$

Even if the Flory–Rehner approach usually applies for mono-polymer systems, it has also been used in bipolymer hydrogel systems where there is significant interaction between the polymer components (i.e., where crosslinking occurs) [32], thus the values presented herein for χ, *M*_c_ and *ν* can be discussed comparatively, considering a “global” contribution of the polymer phase. 

A useful parameter in describing the swelling and drug release from the physically crosslinked hydrogel networks is the mesh size, ξ. For PVA, which is the majoritarian polymer component in the hydrogels, the mesh size could be estimated using Equation (6) [33]:

(6) $$\xi =\Phi_{s}^{-1/3}{\left[C_{\infty }\cdot \left(\frac{2M_{c}}{M_{r}}\right)\right]}^{1/2}\cdot l$$

In Equation (6), *C*_∞_ represents the Flory characteristic ratio (for PVA, *C*_∞_ = 8.4), *M_c_* is the molecular weight between crosslinks (calculated with Equation (3)), *M*_r_ is the molecular mass of the PVA monomer unit (44.05), and *l* is the carbon–carbon bond length of the monomer unit (1.54 Å) [33].

#### 2.3.3. Controlled Release and Adsorption

Similar to the approach described in Section 2.3.2, the tetracycline hydrochloride, amoxicillin, and gentamicin-sulfate-loaded hydrogels corresponding to the formulations depicted in Table 1 were cut into circular disks, weighed (*m*_l_) and immersed into 100 mL of distilled water, respectively, and 100 mL of aqueous media with different pH values (3; 6.5, and 7.3) at room temperature (21 °C) under constant magnetic stirring (50 rpm), to ensure a constant concentration gradient between the gel and the release medium. The pH values were chosen to mimic as close as possible the pH of the gastric fluid (1¨C3), small intestine (6.37–7.04), large intestine (6.63–7.49), and physiological fluid (7.32¨C7.42) [34]. For pH = 7.3, the drug release profiles were studied at different temperatures: 29 °C, 33 °C, and 37 °C.

At predetermined release periods, aliquots were extracted from the release media and analyzed spectrophotometrically (Spekol 11 spectrophotometer, Carl Zeiss, Jena, 0.995 cm glass cuvettes) to determine the amount of released antibiotic (*m*_rel_) at different wavelengths, specific to the determination method for each type of drug, based on prior-constructed calibration curves and taking into account the volume of the release medium, i.e., 100 mL. Tetracycline hydrochloride was determined based on absorbance values measured at λ_max_ = 356 nm [35]. Amoxicillin was determined based on its colored condensation adduct with ascorbic acid absorbing at λ_max_ = 410 nm, according to the procedure described by El-Shafie et al. (limit of detection of 6 ± 0.1 μg/mL) [36]. Gentamicin sulfate was determined according to the method described by Ismail et al., based on the absorbance maximum λ_max_ = 418 nm of gentamicin: ninhydrin complexes (limit of detection of 16 ± 0.3 μg/mL) [37].

The cumulative fractional drug release (*F_t_*) was calculated as the ratio between the amount of released antibiotic *m_rel_* and the initial amount of drug from the hydrogel (*m_in_*, according to Equation (7)) at release time *t* = 0.

(7) $$m_{in}=\frac{m_{l}\cdot \mathrm{CS}\cdot 0.250}{100}\;\left(g\right)$$

Each controlled-release experiment was performed in triplicate, and the average value is presented in the article.

#### 2.3.4. Morpho-Structural Characterization

The nature of the interactions between poly (vinyl alcohol) and ι-carrageenan were assessed performing refraction index ($n_{D}^{25}$) measurements at 25 °C (Abbe refractometer three determinations average) for stock solutions of 1 g polymer/dL of PVA, CAR, respectively, various PVA:CAR mixtures with a variation in carrageenan weight fraction *w_CAR_* (relative to the total amount of polymer in each system) from 0.01 to 0.023 The *w_CAR_* fractions mentioned in Table 1, used to prepare the hydrogels were also included in the study.

A Cannon-Ubbelohde dilution capillary viscometer (viscosimeter constant of 0.0023 cSt/s) was used to determine the relative kinematic viscosity *η_rel_*_,_ and the specific viscosity η_sp_ = (η_rel_ − 1) of the pure polymer solutions and polymer solution mixtures with different *w_CAR_* fractions at 25 °C. To overcome the typical behavior of polyelectrolyte polymers (increase of the reduced viscosity with decreasing concentration, due to stronger electrostatic intramolecular repulsions at lower concentration values), the ι-carrageenan and PVA:CAR mixture solutions were prepared in a sodium phosphate buffer (pH = 6.7) [38]. The dilutions inside the viscometer were performed with the same buffer solution.

For PVA and PVA:CAR mixtures, dilutions of concentration *c_v_* between 0.1 and 0.9 g/dL were employed, obtained from 1 g/dL stock solutions directly inside the viscometer. For ι-carrageenan, a 0.04¨C0.45 g/dL interval was used for *c_v_*. The reduced kinematic viscosity (η_sp_/c_v_) for the polymer solutions was calculated for each concentration. Five measurements were performed for each viscosity value and the average values were presented in the paper. The dependence of η_sp_/c_v_ = f(c_v_) was fitted with the Huggins equation (Equation (8)) [39].

(8) $$\frac{\eta_{\mathrm{sp}}}{c_{v}}=\left[\eta \right]+K_{H}{\left[\eta \right]}^{2}\cdot c_{v}$$

In Equation (9), [η] represents the intrinsic viscosity (dL/g) of the polymers and polymer mixtures and *K_H_* is the Huggins parameter. For flexible polymer macromolecules, *K_H_* value usually lies between 0.2 and 0.8, and favorable polymer–solvent interactions occur. Particularly, if *K_H_* > 1, intermolecular association and entanglement are usually expected [40].

*T*he viscosity interaction parameter *b* (dL^2^/g^2^) is related to the Huggins parameter K_H_ with the following relation (Equation (9)) [39]:

(9) $$b\;={\;K}_{H}\cdot {\left[\eta \right]}^{2}$$

A Quanta FEG 250 scanning electron microscope (SEM) was used to obtain the micrographs of the hydrogels at an acceleration voltage of 20 kV. For SEM analysis, the gels were chemically dehydrated in ethanol according to the procedure detailed in our previous publication [28], to preserve their pore structure as much as possible. A polarized light microscope (RXLr-2Pol, Radical, Ambala, India) was used to visualize the micrographs of the dried gels under cross-polarization conditions.

To supplement the information provided by the SEM micrographs, the pore volume ratio (*Pr*, %) was determined based on the cyclohexane uptake of the dried gels, according to the worksteps described by Jain and Kumar, and our previous research [28,41].

A Fourier transform infrared spectrometer equipped with a total attenuated reflectance device (ATR-FTIR, Nicolet iS10, Thermo Fisher Scientific, Waltham, MA, USA) was used to attain the IR spectra of the samples (dried over CaCl_2_ for seven days before analysis) in the 4000–600 cm^−1^ domain, with a 4 cm^−1^ resolution, with 10 averaged scans per spectrum.

The X-ray diffraction (XRD) spectra of the samples were performed using a Bruker D8 diffractometer (Billerica, MA, USA, Cu K_α_ radiation source at 0.1542 nm), at a scanning speed of 0.04°s^−1^ and a 2θ = 10–40° Bragg angle interval.

The nature of the polymer-drug interactions was studied by electrical conductance measurements on various PC12: antibiotic aqueous solutions with different pH values. For these measurements, similar in principle to conductometric titrations, a starting 1 g polymers/dL solution was used, corresponding to the PC12 formulation, with pH values of 3 and 7.3. Antibiotic solutions of 0.20 g/L concentration, with the same pH values (3, respectively, 7.3) were added stepwise in aliquots of 100 μL to 50 mL of the polymer solution. After each addition, the solutions were magnetically stirred at 100 rpm for 10 min, following the measuring of the electrical conductance (*Q*, mS/cm) with a HI2550 conductometer (Hanna Instruments, Cluj-Napoca, Romania).

The molecular simulations were performed in Materials Studio 7.0 (Accelrys Software Inc., San Diego, CA, USA), using the Amorphous Cell module and the COMPASS forcefield. For modelling ι-carrageenan, 12 repeating (1->3)-β-D-galactopyranose-4-sulfate-(1->4)-3,6-anhydro-α-D-galactopyranose-2-sulfate- segments were used (2 molecules per cell). The poly (vinyl alcohol) was built having 10 repeating units/molecule (isotactic, 4 molecules per cell). The obtained structures were optimized with the same Amorphous Cell module.

## 3. Results and Discussion

### 3.1. Hydrogels Swelling 

**Figure 2:**
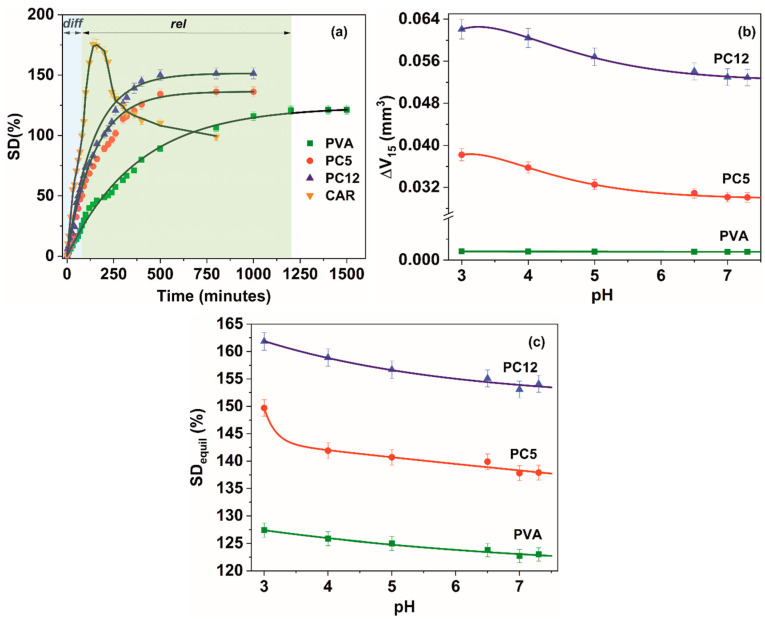
(**a**) Hydrogels swelling kinetic in distilled water; (**b**) variation of the volume of the hydrogel after 15 min of swelling; (**c**) variation in the equilibrium swelling degree with pH at 21 °C (*diff*: diffusional swelling step; *rel*: relaxation swelling regime).

The swelling kinetics of the hydrogels (Figure 2a, for distilled water as swelling medium) consists of two interdependent steps, namely water diffusion into the crosslinked polymer matrix, respectively, relaxation-induced swelling. Water diffusion occurs to a great extent until a swelling degree of ~23¨C47%, having as result a large-scale relative segmental motion of the PVA and/or ι-carrageenan macromolecular chains. After this purely diffusional step threshold, solvent penetration is accompanied to a large extent by dissipation of the strain induced on the physically crosslinked network (viscoelastic relaxation-induced swelling regime) [42,43].

Increasing of the ι-carrageenan amount determines higher equilibrium swelling degrees, due to an increase in the density of the hydrophilic -OH groups from the polymer network. The carrageenan hydrogel (*w_CAR_* = 1) is not stable in the considered aqueous media, significant mass loss occurring after ~5–200 min of immersion, due to the low gel content of this type of hydrogel (Table 1).

Even if the sulfate groups of carrageenan are completely ionized at pH values greater than 2.8, a variation in pH in the 3 ÷ 7.3 domain determines the occurrence of a net osmotic pressure between the inner and the outer environment of the hydrogel. This difference (more significant at pH values of three and four) determines molecular restructuration, modifying the free volume accessible to penetrant water molecules and leading to an increase in swelling (Figure 2b, modification in gel volume) and implicitly in the equilibrium swelling degree (Figure 2c).

The swelling data is in accordance with the crosslinking density (ν) and the molecular weight between crosslinks (M_c_) presented in Table 2. It can be seen that ι-carrageenan (having a higher molecular weight compared to PVA) interferes with the freeze–thaw-induced crosslinking mechanism of PVA, due to the formation of PVA-carrageenan interpolymer complexes, decreasing the crosslinking density, respectively, increasing the molecular weight between the crosslinks, leading to an increase in water swelling. Additionally, the PVA–water interaction parameter χ increases in the blends with the increase in the ι-carrageenan content, implying stronger interpolymer interactions in favor of polymer-water interactions [44]. These poly (vinyl alcohol) ι-carrageenan interactions are proven by the structural data presented in Section 3.3.

To quantitatively assess the diffusional mechanism of water into the PVA, CAR, and PVA:CAR hydrogel matrices, the semiempirical Korsmeyer–Peppas model was applied (Equation (10)) [45].

(10) $${\mathrm{SD}}_{\mathrm{diff}}={\;k}_{D}\cdot t^{n}$$

In Equation (10), SD_diff_ represents the swelling degree for the first 40% of the swelling kinetic, *k*_D_ is the diffusion rate and *n* is the diffusional exponent, which characterizes the type of diffusion. A value of *n* = 0.5 indicates Fickian diffusion, 0.5 < *n* < 1 indicates anomalous (non-Fickian) diffusion, *n* = 1 implies a case II (time-independent) transport, while *n* > 1 implies a super case II transport. 

The portion of the swelling kinetic governed by macromolecular relaxation was fitted with the Hopfenberg model, for the remaining 60% of the swelling kinetic data, until reaching equilibrium (Equation (11)) [46,47].

(11) $$\mathrm{SD}\left(\%\right)={\;\mathrm{SD}}_{\mathrm{diff}}+{\mathrm{SD}}_{\mathrm{rel},\infty }\cdot \left[1-e^{-\kappa \left(t-t_{0}\right)}\right]$$

In Equation (12), the SD_rel,__∞_ represents the equilibrium swelling degree (maximum swelling achieved in the relaxation process), *κ* is the relaxation rate constant, and *t*_0_ is the initial time for the relaxation process.

The effective diffusion coefficients (*D*) of water into the hydrogel matrix were calculated with Equation (12) [31,48]:

(12) $$D\;=\;\pi \cdot {\left(\frac{\delta \cdot \theta }{4\cdot q_{\mathrm{eq}}}\right)}^{2}$$

where δ is the initial thickness of the hydrogel (Table 1), *q*_eq_ represents the ratio between the mass of the swollen hydrogel at equilibrium and its initial mass (at the beginning of the swelling process), and θ is the slope of the swelling kinetic for the first 40% of data.

The data obtained through fitting the swelling kinetic with the Korsmeyer–Peppas and the Hopfenberg models are presented in Table 3.

The data from Table 3 implies that in the case of all hydrogels and respectively, all of the swelling media, the water diffusion is non-Fickian (0.5 < *n* < 1). The relaxation mechanism dominates swelling of all hydrogels, ι-carrageenan addition decreasing the time *(t*_e_) allotted to the purely diffusional step. The prevalence of the relaxation mechanism over the diffusional one has as a consequence a “milder” antibiotic release profile from the polymer matrix (as it can be observed from Section 3.2), proving of higher benefit for sustained, controlled delivery devices. Additionally, the interactions between the poly (vinyl alcohol) and ι-carrageenan are responsible for the decrease in the water uptake rates (κ) registered for the relaxation swelling step. The water diffusion coefficients increase with the addition of ι-carrageenan, with up to 17% for the PC12, in comparison with PVA. The water diffusion coefficients decrease exponentially with the increase of pH, due to intense molecular reorientation occurring at pH values of three and four.

For the hydrogel containing κ-carrageenan (PC12K), in comparison with PC12 (containing ι-carrageenan) the swelling degrees are 10–15% higher. The increase in swelling rates could be due to a looser packing of the macromolecular PVA:CAR assembly (κ-carrageenan has only one sulfate group per repeating unit, thus presenting a lower susceptibility for inter- and intramolecular interactions, i.e., lower gel contents, coupled with a lower crosslinking density ν and mesh size ξ, as seen in Table 1 and Table 2). On a diffusional level, hydrogels with κ-carrageenan have a higher susceptibility to molecular relaxation, with both the swelling rates (*k_D_* and κ) being 50–75% higher than in the case of ι-carrageenan. These higher swelling rates are responsible, in principle, for a higher release rate from the polymer matrix.

### 3.2. Controlled Release from the Hydrogels

The release of the three antibiotics from the polymer matrix is governed by structural factors (molecular mass and hydrodynamic radius of the drug, respectively, the mesh size and swelling of the crosslinked hydrogel matrix), and by the electrostatic interactions between the drug and the ionized sulfate groups of ι-carrageenan, which are dictated by the pH value [49].

**Figure 3:**
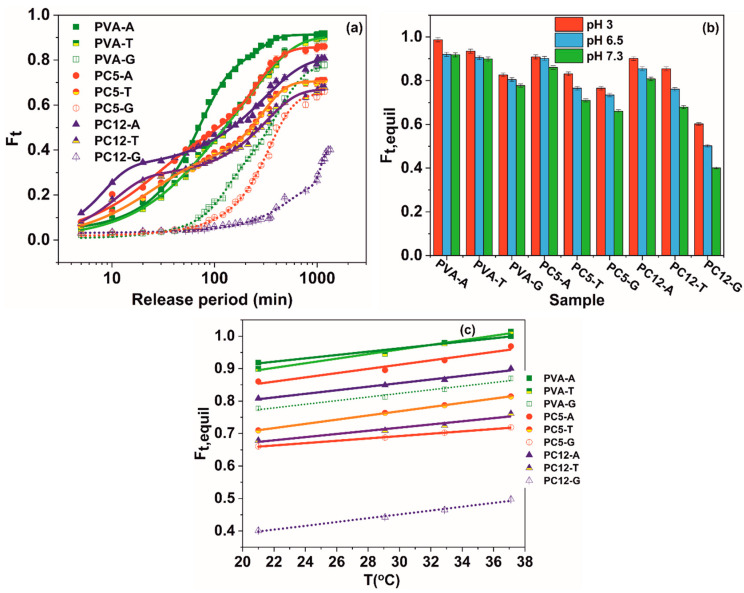
Tetracycline hydrochloride-, amoxicillin-, and gentamicin-sulfate-loaded hydrogels release; (**a**) release kinetic at pH = 7.3 and 21 °C; (**b**) fraction of released antibiotic at equilibrium for different pH values at 21 °C; (**c**) fraction of released antibiotic at equilibrium variation with swelling fluid temperature at pH = 7.3.

Similar to the swelling degree, the antibiotic release occurs at a higher rate in the diffusion-controlled timeframe (until *t*_e_), after which a slow ongoing release occurs in the relaxation domain (Figure 3a).

The antibiotic release profiles were modeled with the Peppas–Sahlin equation (Equation (13)) [50]:

(13) $$\frac{F_{t}}{F_{t,\mathrm{equil}}}={\;k}_{1}\cdot t^{n}+k_{2}\cdot t^{2n}$$

In Equation (13), *F*_t,equil_ signifies the fractional drug degree at equilibrium (Figure 3b), *k*_1_, the antibiotic release rate in the diffusional step, *k*_2_, is the release rate in the swelling-controlled relaxational step and *n* is the diffusional exponent, having the same significance as in Equation (10) (solvent diffusion).

The diffusion coefficients for the period of diffusional release (*D*_diff_), respectively, for the relaxation-controlled release (*D*_rel_) were calculated from the slopes of the linear dependencies derived from Equations (14) (representing F_t_/F_t,equil_ as a function of $t^{1/2}$) and (15) (representing ln (F_t_/F_t,equil_) as a function of t) [32,51]. The obtained values are expressed in Table 4.

(14) $$\frac{F_{t}}{F_{t,\mathrm{equil}}}=4\sqrt{\frac{D_{\mathrm{diff}}\cdot t}{\pi \cdot \delta^{2}}}$$

(15) $$\frac{F_{t}}{F_{t,\mathrm{equil}}}=1-\left(\frac{8}{\pi^{2}}\right)\exp\left[\frac{-\pi^{2}\cdot D_{\mathrm{rel}}\cdot t}{\delta^{2}}\right]$$

Increasing the ι-carrageenan to poly (vinyl alcohol) ratio in the hydrogels determines an increase in the drug release ratio for the first diffusion-controlled step for all three antibiotics (tetracycline hydrochloride (M_w_ = 444.4; hydrodynamic radius of 7.98 nm, pKa_1_ = 3.3, pKa_2_ = 7.2 at 25 °C) [52], amoxicillin (M_w_ = 365.4; hydrodynamic radius of 6.507; pKa_1_ = 2.69; pKa_2_ = 7.3 at 25 °C) [53], and gentamicin sulfate (M_w_ = 516.6; hydrodynamic radius of 9.58 nm; pKa_1_ = 10.18; pKa_2_ = 12.55 at 25 °C) [54], due to the higher solvent penetration rate (*k_D_* values, Table 3).

For the PVA hydrogel, which does not contain ionizable groups, the antibiotic release is completely influenced by swelling, which is pronounced at pH = 3.

For all hydrogels and release media pH, the release rates (*k*_1_ and *k*_2_) and diffusion coefficients (*D*_diff_ and *D*_rel_) decrease in the order amoxicillin > tetracycline hydrochloride > gentamicin sulfate, with increasing of the molecular mass and hydrodynamic radius of the antibiotic. For gentamicin sulfate, its hydrodynamic radius is comparable to the mesh size of PVA (ξ = 13.97 nm, Table 2), which explains the lowest release rate and diffusion coefficient of this drug for the PVA hydrogel, in comparison with the PVA/CAR blends. Due to the macromolecular architecture flexibilization and restructuration promoted by the increase in the release fluid’s temperature, higher cumulative release ratios are registered for the drug-loaded hydrogels at equilibrium (Figure 3c).

**Figure 4:**
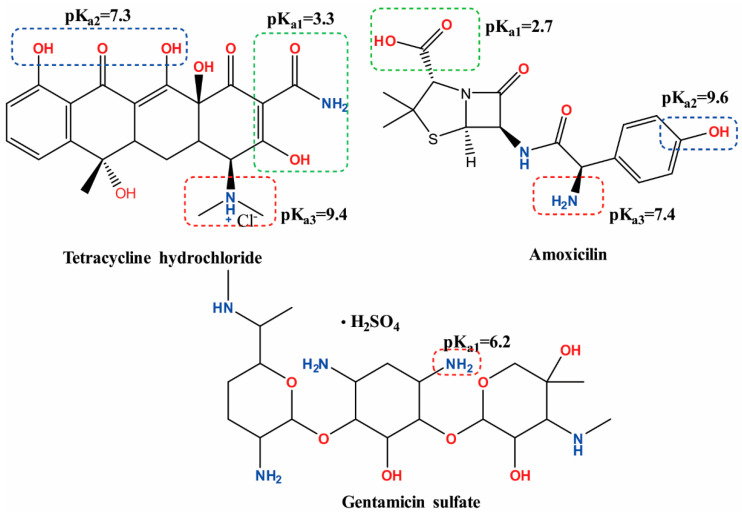
Molecular structure and pKa values for tetracycline hydrochloride, amoxicillin, and gentamicin sulfate.

The molecular structure of the model antibiotics, depicting their pKa values, are presented in Figure 4.

At pH = 3, the carboxyl groups of amoxicillin (pKa_1_ = 2.7) are completely ionized [51], which accounts for higher release rates and diffusion coefficients at low pH values (electrostatic repulsion between the –COO– from amoxicillin and the –OSO_3_^−^ groups of carrageenan). The higher the carrageenan content in the hydrogels, the higher the release rate for amoxicillin at pH = 3. At higher pH values (6.5 and 7.3), increasing of the carrageenan amount has an opposite effect. The amino groups become ionized in higher numbers, leading to a decrease with up to 40% in the release rates for this drug at pH = 7.3, compared with pH = 3. For the same pH value, a variation of 15% in the amoxicillin diffusion rates can be registered between the PC12 blend hydrogel and PVA.

For tetracycline hydrochloride, the conjugated trione system (involving the amide group) is responsible for pKa_1_ = 3.3 [52]. The weakly basic conjugated phenolic enone system becomes involved at pH = 7.3, owing for a 25% decrease in the value of the tetracycline diffusion coefficients, compared to pH = 3. The amino groups become ionized only at pH > 8, which is well outside the pH thresholds for the human body.

The κ-carrageenan hydrogels loaded with tetracycline hydrochloride (PC12K) present higher release rates (with 30–48%) and diffusion coefficients (with 47¨C55%) in comparison with the corresponding ι-carrageenan gels. These release parameters have a much lower variation for PC12K with the pH of the release medium (up to 27%) than in PC12 (up to 47%). Even if the sulfate groups in both carrageenans are completely ionized in the studied pH range, ι-carrageenan seems more sensible to the modifications in the ionic strength of the release medium. These modifications lead in principle to the reorientation in the macromolecular structural assembly, which is responsible for creating new diffusional pathways for the model drug.

The amino and methyl-substituted amino groups of gentamicin become protonated at pH > 7.5 (except for the amino group linked to the third carbon atom from the central streptidinic moiety) [53], so for this drug, the strong hydrogen bonding with PVA and ι-carrageenan (especially since the mesh size of the hydrogels is close to this antibiotic’s hydrodynamic radius) could be responsible for its lower release rates and lower diffusion coefficients, in comparison with the other two antibiotics. The combined diffusional-relaxation mechanisms owe for an anomalous type of release of the antibiotics from the hydrogels (*n* values range from 0.52 to 0.60 for all experimental instances).

### 3.3. Gels Structure and Morphology

**Figure 5:**
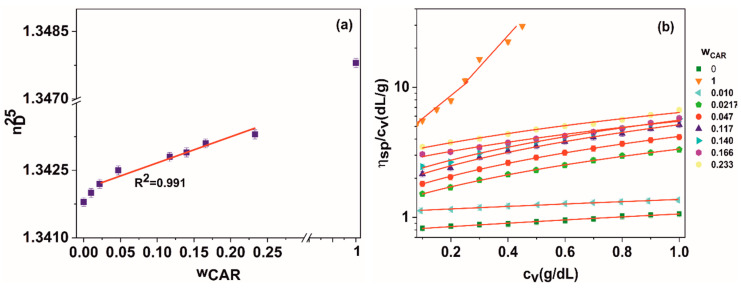
(**a**) Refraction index variation with ι-carrageenan weight fraction for different PVA:CAR mixtures at 25 °C in distilled water (1 g/dL concentration); (**b**) reduced viscosity variation at 25 °C for different PVA:CAR mixtures in phosphate buffer solution.

As it can be seen from Figure 5a, the refraction index variation with ι-carrageenan weight fraction in the 0.02 ÷ 0.23 interval indicates a linear dependency, which implies good miscibility between the two polymers in aqueous solution [55]. The same behavior has been reported for other blends of poly (vinyl alcohol), such as with κ-carrageenan [56] or chitosan [57].

To quantify the miscibility between poly (vinyl alcohol) and ι-carrageenan, the interaction parameter *μ* was calculated (Equation (16)), based on the values of the interaction parameters (*b*) for the blends, respectively, for the pure components (*b*_CAR_, *b*_PVA_), and the intrinsic viscosities of PVA ([η]_PVA_ = 0.793 dL/g), respectively, ι-carrageenan ([η]_CAR_ = 3.816 dL/g), obtained from fitting the dependencies from Figure 5b with the Huggins equation (Equation (9)) [55]:

(16) $$\mu \;=\frac{b-\bar{b}}{2\left(w_{\mathrm{PVA}}\cdot w_{\mathrm{CAR}}\right)\cdot {\left([\eta {]}_{\mathrm{CAR}}-[\eta {]}_{\mathrm{PVA}}\right)}^{2}}$$

where *w*_CAR_ and *w*_PVA_ = 1-*w*_CAR_ represent the weight fraction of CAR, respectively, PVA in the blend, and $\bar{b}$ represents the ideal interaction parameter, calculated according to Krigbaum and Wall with the following expression (Equation (17)) [57]:

(17) $$\bar{b}={\;w}_{\mathrm{PVA}}^{2}\cdot b_{\mathrm{PVA}}+w_{\mathrm{CAR}}^{2}\cdot b_{\mathrm{CAR}}+2w_{\mathrm{PVA}}w_{\mathrm{CAR}}\cdot b$$

The polymer blend is miscible if μ ≥ 0, and immiscible if μ < 0. The interaction parameter values for all blends are given in Table 5. The values of R^2^ correspond to the goodness of the reduced viscosity dependence on *c_v_* being modeled with the Huggins equation (Equation (9)).

For weight ratios of carrageenan higher than 0.01, there is excellent compatibility between the two polymers (μ > 0). Since ι-carrageenan presents a higher molecular mass than PVA, at sufficiently higher CAR/PVA ratios, it could act as a pseudotheta solvent for PVA, forming rigid polymer intercomplexes by entangling around the shorter flexible PVA chains. These intercomplexes disrupt the original rigid double-helix conformation of ι-carrageenan (K_H_ value of carrageenan is greater than (1) flexibilizing it; therefore, the PVA macromolecules could function as a pseudoplasticizer for the rigid carrageenan macromolecules, a role which seems to be confirmed for the lowering of *K_H_* values of the blends at increased carrageenan content. For low carrageenan amounts (*w*_CAR_ = 0.01), phase separation occurs (μ < 0) due to a more reduced possibility of chain entanglement. which may lead to miscibility between the two polymers. The compositions for the two PC5 and PC12 hydrogels correspond to high values of μ, leaving out the possibility of forming two distinct polymer-rich phases during cryogelation.

**Figure 6:**
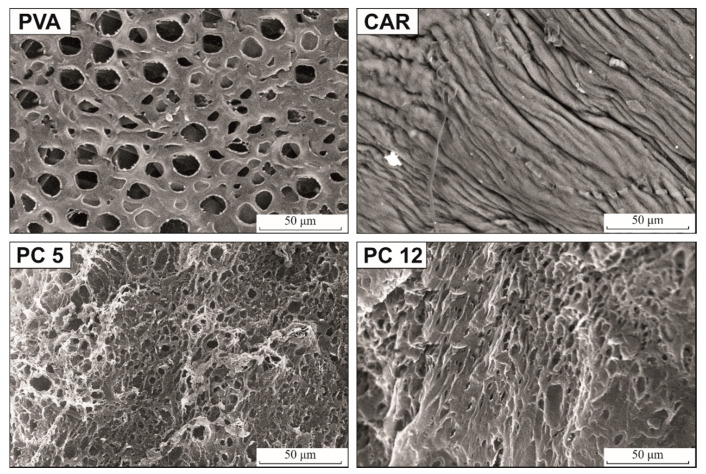
SEM micrographs depicting the surface morphologies of the obtained hydrogels.

A smooth and homogeneous morphology can be observed for the PVA hydrogel, with macropores ranging from 8 to 15 μm (Figure 6). In contrast, for the ι-carrageenan hydrogel surface, no apparent porosity was observed, implying a different gelation mechanism for this polymer, in contrast to PVA. In PVA solutions, freezing induces the formation of two bicontinuous phases: a polymer-rich swollen phase in which crystallites (i.e., crosslinking points) are formed, and a free freezable water-rich phase, which shapes the network’s pore morphology [58]. Carrageenan, like other hydrocolloids, forms freestanding thermoreversible gels above a critical concentration value, when the macromolecules come in close proximity with one another, forming weakly associated regions (junction zones), which are responsible for the very low value of the gel content (Table 1) [59]. The critical concentration value for the ι-carrageenan used in this study could be determined as the inflection (slope-changing) point in the reduced viscosity variation with concentration [60] from Figure 5b (*c*_crit_ = 0.26 g/dL), so the solutions containing 2 wt/vol % ι-carrageenan will have a high tendency to gel.

The addition of ι-carrageenan to PVA determines a decrease in the pore size and density of the PC5 and PC12 hydrogels, as also determined from the *Pr* values from Table 6, calculated from the cyclohexane uptake values. A more compact surface can be observed with increasing carrageenan content, probably due to a substantial decrease in the free water content in the gelling systems and to the stabilization effect of CAR, which interferes with the phase separation process during freezing. The decrease in pore size with the increasing amount of polysaccharide has also been observed for PVA/chitosan blend hydrogels above 40 wt % chitosan [61], for PVA/hydroxyethyl starch [62] or PVA/gelatin [63] hydrogels prepared through cryogelation. Even if the water diffusion coefficients increase proportionally with the ι-carrageenan content, the equilibrium swelling values do not increase following the same trendline, because the reduced porosity provides less diffusional pathways for water penetration into the hydrogel network, and retards the release of antibiotics from the hydrogel in the relaxation step.

**Figure 7:**
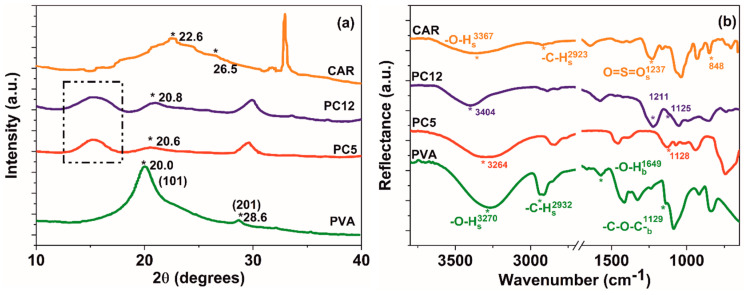
(**a**) X-ray diffraction patterns and (**b**) ATR-FTIR spectra of the obtained hydrogels (s: stretching; b: bending vibrations).

ι-carrageenan determines only a slight decrease in the crystallinity (*Cr*^XRD^) of the hydrogel blends, as shown in Table 6. The (101) and (201) reflections, corresponding to the monoclinic unit cell of PVA [64] (Figure 7a) shift to higher 2θ numbers in the PC5 and PC12 blends, implying a reduction in crystallite sizes (*D*, Table 6, determined with the Debye–Scherrer equation [28,65]), possibly due to a tighter packing of the ordered macromolecular segments dictated by increased interaction through hydrogen bonding between PVA and CAR. This behavior has also been observed for PVA/κ-carrageenan or PVA/chitosan blends.

Additionally, a weak diffraction hallo could be seen for PC5 (15.3°), respectively, PC12 (15.2°), which is shifted to lower diffraction angle values comparing with the broad reflection centered at 22.6° for CAR, accounting for the contributions of the amorphous macromolecular regions. The blue-shifting of this diffraction contribution could imply the formation of chain entanglements between PVA and ι-carrageenan in the amorphous macromolecular domains. This behavior has also been documented for PVA/chitosan [66] and PVA/cellulose [63] hydrogels. 

The FTIR spectra of the PC5 and PC12 blends from Figure 7b indicate that poly (vinyl alcohol) and ι-carrageenan interact through hydrogen bonding. The appearance of new absorption bands signaling covalent bonding was not observed for this blend.

**Figure 8:**
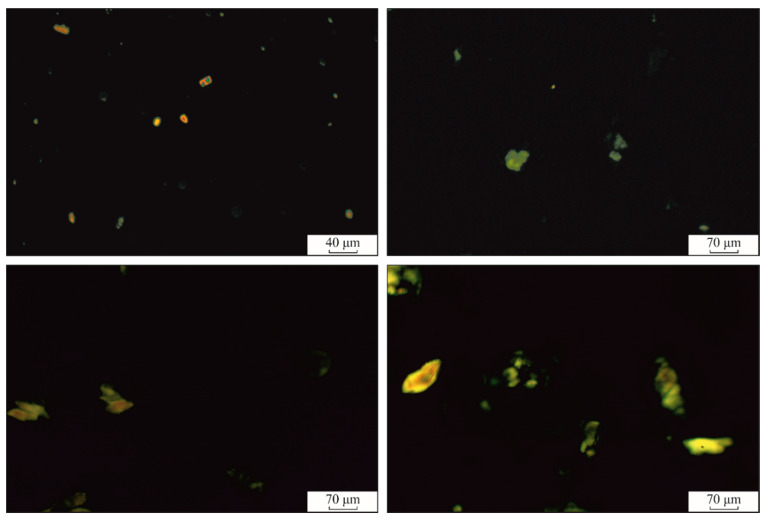
Cross-polarized microscopy of the hydrogels surface (magnification: 10× for CAR and 4× for PVA, PC5, and PC12).

**Figure 9:**
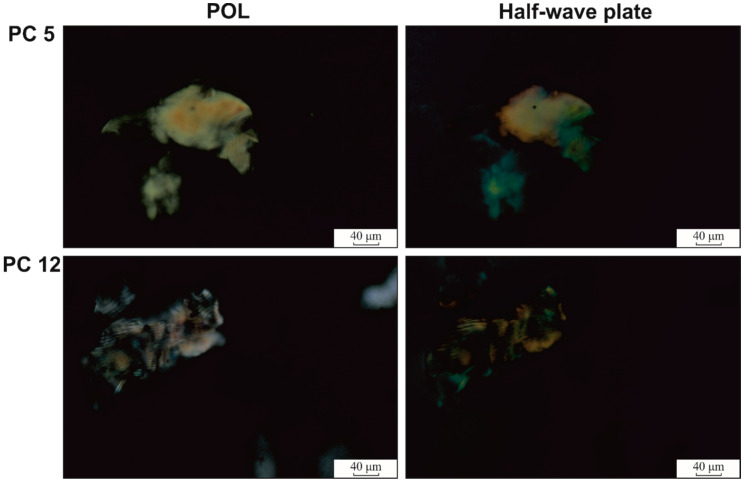
Cross-polarized and λ/2 quartz wave plate microscopy of the surface of the hydrogel at 10× magnification (POL: micrographs acquired under cross-polarization regime).

The crystallites could be associated with aggregates uniformly embedded in the amorphous macromolecular domains and are visible in the cross-polarization optical micrographs of the hydrogels (Figure 8 and Figure 9). In the case of ι-carrageenan, these associations have an average diameter of 7 μm and seem to be in a lower amount than in PVA, according to its low crystallinity value.

Increasing the content of ι-carrageenan determines the formation of crystalline regions with higher dimensions (85 μm average diameter for PC5 and 120 μm for PC12), compared to PVA and CAR. This behavior seems to confirm the macromolecular entanglement of the two components. Since the crystalline domains act as junction regions and spacers, basically defining the hydrogels’ network, the larger the individual junction regions, the higher the water sorption capacity.

The half-wave polarized microscopy images confirm the crystalline nature of the aggregates observed from Figure 9, where these domains present birefringence. A closer look at these aggregates reveals parallel-aligned fringes, separated by darker (amorphous) domains.

Interaction through inter- and intramolecular hydrogen bonds could be deducted from the shifting of the PVA crystallinity band (1129 cm^−1^ (PC5 and PC12) [67], and of the ι-carrageenan O=S=O sulfate stretching vibration (1237 cm^−1^, for PC12) to lower wavenumbers (Figure 7b) [68]. The crystallinity index of PVA (*CrI*^FTIR^), calculated as the ratio between the height of the bands centered at 1128 and 1089 cm^−1^ [67] is found to be proportional to the crystallinity calculated from the XRD diffractograms (*Cr*^XRD^, Table 6), behavior similar to that of PVA/κ-carrageenan system. Another indication of an enhanced interaction between PVA and CAR is the hydrogen bond energy (*E*_H_), which is calculated according to our previous work [28]. Increased value of hydrogen bond energy for PC5, compared to the values for PVA or CAR implies a stronger interaction between these components, higher water stability, and a decrease in the diffusion coefficients of active species from the hydrogel matrix when in swelling relaxation regime.

In drug delivery, polymer matrix-guest molecule interactions are determinant for the release profiles. For PVA, diffusion is controlled by the chemistry of the release medium (ionic strength and nature of the present chemical species (kosmotropes, chaotropes)), which enhances or disrupts the hydrogen bonding in the hydrogel. Supplementary, by adding ι-carrageenan to PVA additional electrostatic interactions could be promoted between the completely ionized sulfate groups and the antibiotics, as a function of the pH of the release medium.

**Figure 10:**
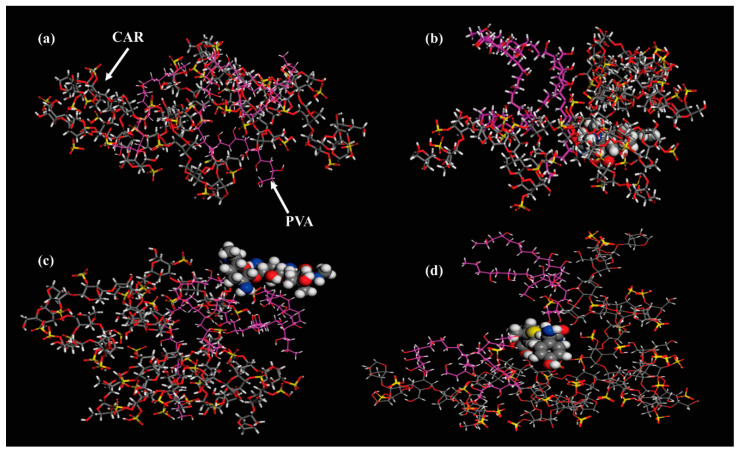
Energy-minimized molecular simulation snapshots of (**a**) PVA: CAR; (**b**) PVA: CAR: tetracycline hydrochloride; (**c**) PVA: CAR: gentamicin sulfate; (**d**) PVA: CAR: amoxicillin (the PVA C-C chains are colored in purple, and the antibiotic molecules are designated in each case with space-filling calotte models).

The molecular simulation snapshots of the PVA:CAR system (Figure 10) sustain the discussion related to the interaction parameters (Table 5). It can be seen from Figure 10a that the lower molecular weight PVA chains entangle around the ι-carrageenan molecular assemblies, but are not preferentially intercalated between the ι-carrageenan molecules.

**Figure 11:**
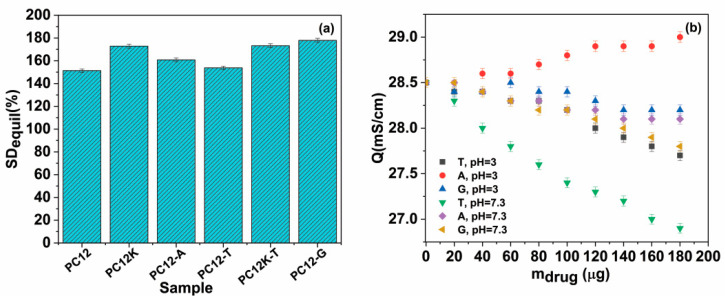
(**a**) Equilibrium swelling values for the PC12, PC12K hydrogels, and their antibiotic-loaded counterparts; (**b**) electrical conductance variation in the PC12: antibiotic aqueous system (the equilibrium swelling values from Figure 10a take into account the mass of released antibiotic; T: tetracycline hydrochloride, A: amoxicillin; G: gentamicin sulfate).

Firstly, all the antibiotics included in the polymer matrix have a “spacer” effect, leading to the increase in the mesh size of the polymer network (mesh sizes ξ for PC12-A, PC12-T, and PC12-G are all higher than the corresponding value registered for PC12). This determines higher equilibrium swelling values for all drug-loaded hydrogels compared to the reference (Figure 11a). This effect is also registered for the hydrogels containing κ-carrageenan, but shifted to higher swelling equilibrium values compared to the gels containing ι-carrageenan.

Tetracycline hydrochloride, through its conjugated trione moiety (δ+), can interact with the sulfate groups of ι-carrageenan (δ−), practically leading to the highest crosslinking density among the drug-loaded hydrogels, but slightly higher than PC12. The molecular simulation snapshot from Figure 10b seems to confirm this statement, as this molecule has the highest intertwining with the polymer phase. For this hydrogel, the lowest equilibrium swelling values were registered.

Gentamicin sulfate, due to the presence of the -NH_2_ groups (δ+) determines the second-highest crosslinking density among the drug-loaded hydrogels (Table 2). Due to its large hydrodynamic radius, this molecule is less efficiently embedded in the polymer phase than tetracycline hydrochloride, being distributed mainly in the PVA-rich phase (Figure 10c). Since the mesh size of PVA is comparable to the hydrodynamic radius of gentamicin sulfate, this could explain the lowest release rate of this drug for all experimental instances studied in this paper. This molecule could act as a “plug”, reducing water the water uptake and swelling of the hydrogel. Amoxicillin, due to its lowest hydrodynamic radius can penetrate more efficiently into the polymer phase (Figure 10d). Due to the ionized (dissociated) carboxyl groups (δ−), amoxicillin promotes the highest spacer effect, repelling the carrageenan molecules, determining the lowest crosslinking density, and the highest water uptake at equilibrium among the drug-loaded hydrogels. 

The electrical conductance variation with the amount of added antibiotic (Figure 11b) reveals that except for amoxicillin at pH = 3 (for which the carboxyl groups are ionized), in all cases a decrease in the overall conductance of the system is registered, signaling a degree of ionic interaction between ι-carrageenan and the antibiotic.

The highest drop is registered for tetracycline hydrochloride. Therefore, even if there are interactions between carrageenan and the model antibiotics, these do not lead to a crosslinking of the polymer matrix in the traditional sense (linking macromolecular chains and lowering the swelling degree of the material). Instead, this interaction (possibly coupled with hydrogen bonding) modulates the drug release patterns and leads in each case to a small “bound” amount of antibiotic embedded in the matrix (Figure 3b) after the release step.

## 4. Conclusions

The addition of ι-carrageenan (which possesses two sulfate groups per galactan unit) to poly (vinyl alcohol) leads to the formation of entirely miscible blends, which were used to obtain hydrogels by cryogelation (applying five alternate freezing and thawing cycles). Poly (vinyl alcohol) and carrageenan are entirely compatible in the amorphous domains, and to a limited extent, in the crystalline domains, forming mixed crystallite associations with lower diameters than in the case of the pure polymers. These traits, alongside the reasonably high gel contents, are responsible for the hydrogels’ excellent stability in aqueous environments with pH values between 3 and 7.3.

The swelling of the polymer blend matrix is governed by relaxation, which determines a retarding in the release of the model antibiotic drugs (amoxicillin, tetracycline hydrochloride, and gentamicin sulfate) from the polymer matrix with values up to 40%, compared to the poly(vinyl alcohol) reference hydrogel at different pH values, depending on the chemistry of the drug.

Due to the presence of the completely ionized sulfate groups, ι-carrageenan determines a more pronounced drug release modulation as a function of pH compared to the reference poly (vinyl alcohol) hydrogel, trough electrostatic interactions, and/or hydrogen bonding.

This study represents an extension of our previous work on poly (vinyl alcohol) and κ-carrageenan hydrogels (which were used as sorbent materials for cationic dyes sorption), aiming to enlarge the database regarding the structure and applicability domains of materials obtained with this type of sulfated polysaccharide.

Comparing ι-carrageenan with κ-carrageenan at the same weight ratio, the former determines up to 15% lower equilibrium swelling degrees due to a tighter packing of the macromolecular assembly. The latter determines a faster release of tetracycline hydrochloride (release rates and diffusion coefficients up to 48% and 55% higher).

## Figures and Tables

**Table 1:** Composition, thickness, solids, and gel contents of the obtained hydrogels *.

Sample Code	Polymeric Components Amount				w_CAR_	m_drug_ (g)	δ (mm)	SC (%)	GC (%)
	PVA		CAR						
	PVA Solution Volume (mL)	PVA Amount (g)	CAR Solution Volume (mL)	CAR Amount (g)					
PVA	10	1.000	–	–	0	0.250	3.87	9.81	87.21
PC5	8	0.800	2	0.040	0.047	0.210	3.81	8.22	82.78
PC12	6	0.600	4	0.080	0.117	0.170	3.74	5.63	83.31
PC12K	6	0.600	4	0.080	0.117	0.170 **	3.79	5.43	79.81
CAR	–	–	10	0.200	1	–	3.62	2.04	0.83

* Maximum relative error: for solids content and gel content: ± 1.1%, and for the thickness δ: ± 0.1%; ** tetracycline hydrochloride.

**Table 2:** Parameters related to crosslinking density (ν), molecular weight between crosslinks (*M_c_*), and mesh size (ξ) for the neat and drug-loaded hydrogels.

Sample Code	ϕ_s_⋅10^2^	χ	ν⋅10^4^	*M*_c_	ζ (nm)
PVA	7.210	0.525	23.700	445.13	13.970
PC5	4.320	0.528	8.091	1295.15	28.270
PC12	2.212	0.530	1.998	5204.83	70.810
PC12K	2.013	0.544	1.274	6378.27	80.312
PC12-A	2.032	0.505	1.650	6729.57	82.980
PC12-T	2.203	0.508	1.980	5630.57	73.770
PC12-G	2.069	0.507	1.730	6439.89	80.601
CAR	1.140	0.504	0.846	11,977.17	–

**Table 3:** Parameters related to the swelling kinetic and water diffusion *.

Material	Parameter	Swelling Medium					
		Distilled Water	pH = 3	pH = 4	pH = 5	pH = 6.5	pH = 7.3
**PVA**	**k_D_ (min^−n^)**	0.42 (0.990)	0.54 (0.996)	0.50 (0.998)	0.47 (0.993)	0.45 (0.994)	0.46 (0.992)
	***n***	0.92 (0.990)	0.97 (0.996)	0.94 (0.998)	0.95 (0.993)	0.99 (0.994)	0.94 (0.992)
	**SD_diff_ (%)**	23.09 (0.988)	21.90 (0.981)	22.49 (0.990)	22.67 (0.987)	22.78 (0.991)	22.97 (0.996)
	**SD_rel,_** **_∞_** **(%)**	122.53 (0.988)	126.45 (0.981)	125.57 (0.990)	124.73 (0.987)	123.98 (0.991)	122.13 (0.996)
	**κ** **× 10^2^ (min^−1^)**	1.66 (0.988)	1.79 (0.981)	1.72 (0.990)	1.71 (0.987)	1.68 (0.991)	1.70 (0.996)
	***t*** **_o_** **(min)**	88.19 (0.988)	79.12 (0.981)	79.83 (0.990)	83.11 (0.987)	85.01 (0.991)	88.04 (0.996)
	***D*** **× 10^5^ (cm^2^/s)**	8.64	10.13	10.04	9.58	8.69	8.72
**PC5**	***k*** **_D_** **(min^−n^)**	0.55 (0.961)	0.68 (0.994)	0.63 (0.989)	0.59 (0.995)	0.57 (0.998)	0.55 (0.990)
	***n***	0.87 (0.961)	0.94 (0.994)	0.92 (0.989)	0.90 (0.995)	0.89 (0.998)	0.86 (0.990)
	**SD_diff_ (%)**	46.21 (0.955)	40.34 (0.994)	41.77 (0.997)	43.11 (0.991)	43.52 (0.994)	46.73 (0.992)
	**SD_rel,_** **_∞_** **(%)**	137.60 (0.955)	149.56 (0.993)	140.34 (0.997)	139.83 (0.991)	138.84 (0.994)	137.97 (0.992)
	**κ** **× 10^3^ (min^−1^)**	5.90 (0.955)	8.31 (0.993)	8.04 (0.997)	7.56 (0.991)	7.00 (0.994)	6.43 (0.992)
	***t*** **_o_** **(min)**	60.91 (0.955)	39.90 (0.993)	45.58 (0.997)	51.78 (0.991)	55.00 (0.994)	58.72 (0.992)
	***D*** **× 10^5^ (cm^2^/s)**	8.90	18.34	16.98	12.65	10.19	9.04
**PC12**	***k*** **_D_** **(min^−n^)**	1.26 (0.966)	1.42 (0.992)	1.38 (0.995)	1.35 (0.988)	1.30 (0.991)	1.29 (0.997)
	***n***	0.80 (0.966)	0.89 (0.992)	0.87 (0.995)	0.85 (0.988)	0.83 (0.991)	0.83 (0.997)
	**SD_diff_ (%)**	27.88 (0.949)	20.11 (0.988)	21.98 (0.994)	22.65 (0.989)	24.77 (0.990)	27.10 (0.992)
	**SD_rel,_** **_∞_** **(%)**	153.08 (0.949)	160.56 (0.988)	158.77 (0.994)	156.75 (0.989)	155.04 (0.990)	154.07 (0.992)
	**κ** **× 10^3^ (min^−1^)**	5.60 (0.949)	7.02 (0.988)	6.72 (0.994)	6.28 (0.989)	5.91 (0.990)	5.67 (0.992)
	***t*** **_o_** **(min)**	29.37 (0.949)	15.34 (0.988)	18.62 (0.994)	22.86 (0.989)	24.11 (0.990)	28.75 (0.992)
	***D*** **× 10^4^ (cm^2^/s)**	1.04	5.87	4.33	2.85	2.07	1.13
**PC12K**	***k*** **_D_** **(min^−n^)**	1.34 (0.998)	1.53 (0.991)	1.50 (0.990)	1.42 (0.989)	1.38 (0.992)	1.35 (0.997)
	***n***	0.93 (0.998)	0.96 (0.991)	0.95 (0.990)	0.94 (0.989)	0.94 (0.992)	0.93 (0.997)
	**SD_diff_ (%)**	22.65 (0.994)	19.83 (0.998)	19.97 (0.995)	20.12 (0.0990)	20.21 (0.996)	22.03 (0.992)
	**SD_rel,_** **_∞_** **(%)**	172.86 (0.994)	181.49 (0.998)	176.42 (0.995)	173.16 (0.990)	173.04 (0.996)	173.21 (0.992)
	**κ** **× 10^2^ (min^−1^)**	2.07 (0.994)	4.09 (0.998)	3.92 (0.995)	3.56 (0.990)	3.28 (0.996)	2.09 (0.992)
	***t*** **_o_** **(min)**	14.78 (0.994)	7.39 (0.998)	8.12 (0.995)	9.77 (0.990)	12.27 (0.996)	12.85 (0.992)
	***D*** **× 10^3^ (cm^2^/s)**	4.07	4.88	4.74	4.51	4.16	4.10
**CAR**	***k*** **_D_** **(min^−n^)**	2.73 (0.991)	n.a. (gel not stable)				
	***n***	0.82 (0.991)					
	**SD_diff_ (%)**	26.85 (0.831)					
	**SD_rel,_** **_∞_** **(%)**	158.71 (0.831)					
	**κ** **× 10^2^ (min^−1^)**	2.20 (0.831)					
	***t*** **_o_** **(min)**	11.15 (0.831)					
	***D*** **× 10^3^ (cm^2^/s)**	3.86					

* Correlation coefficients indicating the appropriateness of the fitting model are given in parenthesis after the value of each parameter.

**Table 4:** Parameters related to the antibiotics release kinetic profiles and diffusion at 21 °C (A: amoxicillin; T: tetracycline hydrochloride and G: gentamicin sulfate) *.

Material	Parameter	Swelling Medium								
		pH = 3			pH = 6.5			pH = 7.3		
**PVA**	**Antibiotic**	**A**	**T**	**G**	**A**	**T**	**G**	**A**	**T**	**G**
	***k*** **_1_** **× 10^2^ (min^−m^)**	7.04(0.993)	6.04(0.995)	3.27(0.997)	5.89(0.992)	4.50(0.992)	2.89(0.994)	5.23(0.992)	4.39(0.996)	2.78(0.997)
	***k*** **_2_** **× 10^3^** **(min^−2n^*)***	7.98(0.993)	7.26(0.995)	2.82(0.997)	7.84(0.992)	6.22(0.992)	2.34(0.994)	7.36(0.992)	6.12(0.996)	1.10(0.997)
	***n***	0.58(0.993)	0.56(0.995)	0.54(0.997)	0.60(0.992)	0.58(0.992)	0.54(0.994)	0.63(0.992)	0.60(0.996)	0.56(0.997)
	***D*** **_diff_** **× 10^6^ (cm^2^/s)**	8.97(0.999)	8.73(0.987)	4.17(0.994)	8.33(0.987)	7.18(0.988)	3.94(0.990)	8.04(0.988)	6.24(0.989)	3.76(0.996)
	***D*** **_rel_** **× 10^6^ (cm^2^/s*)***	4.78(0.988)	4.21(0.989)	1.55(0.989)	4.16(0.988)	4.06(0.991)	1.48(0.986)	4.11(0.991)	3.04(0.998)	1.26(0.994)
**PC5**	***k*** **_1_** **× 10^2^ (min^−m^)**	8.12(0.994)	6.85(0.997)	3.94(0.994)	6.05(0.996)	4.82(0.992)	3.67(0.991)	5.68(0.992)	4.77(0.995)	2.92(0.999)
	***k*** **_2_** **× 10^3^** **(min^−2n^)**	9.04(0.994)	7.08(0.997)	2.78(0.994)	7.02(0.996)	5.56(0.992)	2.28(0.991)	6.98(0.992)	4.01(0.995)	1.02(0.999)
	***n***	0.55(0.994)	0.54(0.997)	0.54(0.994)	0.56(0.996)	0.55(0.992)	0.52(0.991)	0.58(0.992)	0.56(0.995)	0.54(0.999)
	***D*** **_diff_** **× 10^6^ (cm^2^/s)**	9.31(0.992)	8.89(0.991)	4.28(0.992)	8.87(0.990)	7.24(0.991)	4.08(0.993)	8.12(0.991)	6.79(0.989)	3.83(0.998)
	***D*** **_rel_** **× 10^6^ (cm^2^/s*)***	4.82(0.991)	4.12(0.992)	1.31(0.990)	4.06(0.994)	3.51(0.997)	1.24(0.991)	4.02(0.994)	2.97(0.987)	1.13(0.993)
**PC12**	***k*** **_1_** **× 10^2^ (min^−m^)**	8.44(0.998)	6.90(0.992)	3.98(0.996)	6.87(0.998)	5.34(0.994)	3.78(0.998)	5.91(0.994)	4.82(0.991)	2.98(0.988)
	***k*** **_2_** **× 10^3^** **(min^−2n^)**	9.26(0.998)	6.98(0.992)	2.42(0.996)	8.03(0.998)	4.84(0.994)	2.08(0.998)	6.71(0.994)	3.96(0.991)	0.97(0.998)
	***n***	0.53(0.998)	0.53(0.992)	0.53(0.996)	0.54(0.998)	0.53(0.994)	0.53(0.998)	0.56(0.994)	0.55(0.991)	0.52(0.998)
	***D*** **_diff_** **× 10^6^ (cm^2^/s)**	9.38(0.994)	8.96(0.993)	4.36(0.994)	8.90(0.987)	6.91(0.990)	4.11(0.988)	8.24(0.990)	6.83(0.992)	3.91(0.996)
	***D*** **_rel_** **× 10^6^ (cm^2^/s*)***	4.92(0.990)	4.06(0.991)	1.18(0.987)	3.89(0.986)	3.16(0.991)	1.10(0.990)	2.98(0.988)	2.90(0.986)	1.04(0.998)
**PC12K**	***k*** **_1_** **× 10^1^ (min^−m^)**	–	1.02(0.998)	–	–	0.82(0.992)	–	–	0.73(0.998)	–
	***k*** **_2_** **× 10^2^ (min^−2n^)**	–	3.28(0.998)	–	–	2.81(0.992)	–	–	2.46(0.998)	–
	***n***	–	0.61(0.998)	–	–	0.60(0.997)	–	–	0.56(0.998)	–
	***D*** **_diff_** **× 10^5^ (cm^2^/s)**	–	2.18(0.989)	–	–	2.08(0.994)	–	–	1.92(0.993)	–
	***D*** **_rel_** **× 10^5^ (cm^2^/s*)***	–	6.82(0.991)	–	–	6.77(0.996)	–	–	6.21(0.995)	–

* Correlation coefficients indicating the appropriateness of the fitting model are given in parenthesis after the value of each parameter.

**Table 5:** Huggins parameters and interaction parameters for PVA:CAR blends *.

Blend Composition		(η) (dL/g)	Κ_H_	b (dL^2^/g^2^)	μ	R^2^
w_PVA_	w_CAR_					
**1**	**0**	0.793	0.427	0.268	–	0.998
0.990	0.010	1.107	0.214	0.263	−0.02	0.989
0.978	0.021	1.311	1.081	1.860	4.06	0.998
**0.953**	**0.047**	1.552	1.090	2.627	2.81	0.999
**0.883**	**0.117**	1.846	0.965	3.290	1.44	0.996
0.860	0.140	2.026	0.869	3.574	1.31	0.995
0.834	0.166	2.583	0.444	2.968	0.85	0.981
0.767	0.233	3.032	0.437	4.017	0.63	0.986
**0**	**1**	3.816	1.346	19.618	–	0.978

* The composition values presented in boldface correspond to the starting solutions from which PVA, PC5, PC12, and CAR gels were prepared through five successive freeze–thaw cycles.

**Table 6:** Pore volume ratio (*Pr*) and structural parameters for the obtained hydrogels.

Sample Code	*Pr* (%)	Cr^XRD^ (%)	D (nm)	CrI^FTIR^	E_H_ (kcal)	
PVA	8.23	11.03	7.94	0.24	6.12	
PC5	6.04	9.94	7.68	0.21	6.22	
PC12	5.14	7.27	7.57	0.15	3.96	
CAR	0.73	4.05	–	–	4.56	
